# Exploring the Relation Between Coexisting Chronic Lymphocytic Leukemia and Lung Cancer: Two Case Reports

**DOI:** 10.1155/crom/6589796

**Published:** 2025-12-30

**Authors:** Toka Amin, Gowri Swaminathan, Muhammad Haseeb Ul Rasool, Utsow Saha, Jie Huang

**Affiliations:** ^1^ Department of Medicine, Henry Ford Providence Hospital, Southfield, Michigan, USA; ^2^ Department of Pathology, Icahn School of Medicine at Mount Sinai/Queens Hospital Center, New York, New York, USA

**Keywords:** CLL, HER-2/neu, immunocompromised, lung cancer, second primary malignancy, smoking

## Abstract

Chronic lymphocytic leukemia (CLL) is a low‐grade lymphoproliferative disorder characterized by clonal proliferation of lymphocytes. The disease is indolent but might be complicated with life‐threatening cytopenias, high‐grade transformation, or second primary malignancies (SPMs). The incidence of SPMs is higher in CLL compared to the normal population; however, the genetic association and predisposing factors linking CLL to SPMs remain unclear. Synchronous or metasynchronous CLL and lung cancer are relatively uncommon, and the relationship is rarely explored in the literature. We report two cases of a 77‐year‐old male and a 52‐year‐old female with a history of heavy smoking, both presenting with respiratory symptoms. Imaging revealed lung masses, hilar adenopathy, pleural effusions, and visceral metastasis. The first case′s pleural fluid cytology was positive for both CLL and adenocarcinoma, while the second patient was diagnosed with a biopsy from the right supraclavicular mass revealing carcinoma. In both patients, a flow cytometry of the peripheral smear confirmed the diagnosis of CLL. Both patients had a similar outcome of death due to complicated intracerebral metastatic disease with intracranial hemorrhage and vasogenic edema within 1–2 months of diagnosis. Although incompletely understood, CLL and lung cancer share some predisposing social and biological factors, of which smoking is the most evident. It is also thought that the immunocompromised state of CLL patients is a risk factor for SPMs, given qualitative and quantitative defects in almost all immune cell lines. The cytogenetic association has been suggested but has yet to be comprehensively explored. The proposed tumor factors include HER‐2/neu overexpression in lung cancer cells and Trisomy 12 in B‐cell clones, which neither of our patients had. Despite suggested links, the relationship between CLL and lung cancer needs further exploration which might offer a future benefit for anticipation and earlier detection of lung cancer in CLL patients.

## 1. Background

Chronic lymphocytic leukemia (CLL) is a common low‐grade lymphoproliferative disorder characterized by a massive accumulation of Clonal B lymphocytes in peripheral blood and lymphatic organs. The disease course is extremely heterogeneous, and outcomes vary from no indicated treatment to a much poorer prognosis as a result of serious cytopenias, transformation to deadly hematologic malignancies, or development of secondary tumors. CLL patients are at higher risk of having second hematologic or solid malignancies compared to the general population as a result of genetic alterations such as Trisomy 12 or as a complication of cancer therapy [1]. Coexisting lung cancer and CLL are not adequately reported in the literature, and a clear association between the two malignancies is not yet identified [2, 3]. We report two rare cases of double primary CLL and lung cancer sharing similar presentations and outcomes.

## 2. Case Presentation

### 2.1. Case 1

A 77‐year‐old male patient with a past medical history of Type 2 diabetes, hypertension, a former smoker who quit 35 years ago, and a history of thoracentesis for pleural effusion in India 2 weeks before presentation presented to the emergency department with 2 months of progressive shortness of breath, marked fatigue, nonproductive cough, and a left‐sided nonradiating intermittent chest pain that started 4 days before presentation and exacerbated with coughing. His symptoms were associated with decreased appetite and unintentional weight loss of 15–20 kg in 2 months. The physical exam was significant for diffuse anterior cervical and axillary adenopathy, absent breath sounds and dullness on percussion of the left lung, and poor air entry on the right side. Initial labs were remarkable for a white blood count of 142 × 10^3^/*μ*L, with absolute lymphocytes counting for > 100 × 10^3^/*μ*L, hemoglobin of 10.9 g/dL, RBCs of 3.73 × 10^3^/*μ*L, MCV of 100.8 fL, and platelets of 152 × 10^3^/*μ*L. Peripheral smear showed lymphocytosis of small size, round nuclei, clumped chromatin, and inconspicuous nucleoli with no blasts.

**Figure 1:**
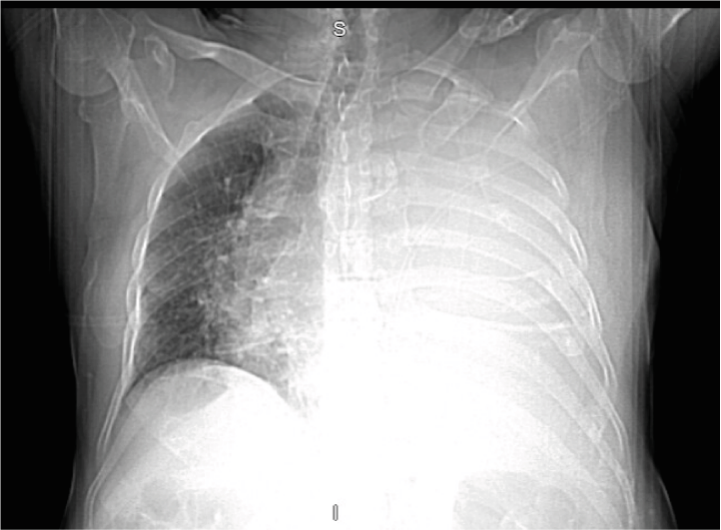
Chest radiograph showing total opacification of the left hemithorax with a shift of the mediastinum to the right side.

**Figure 2:**
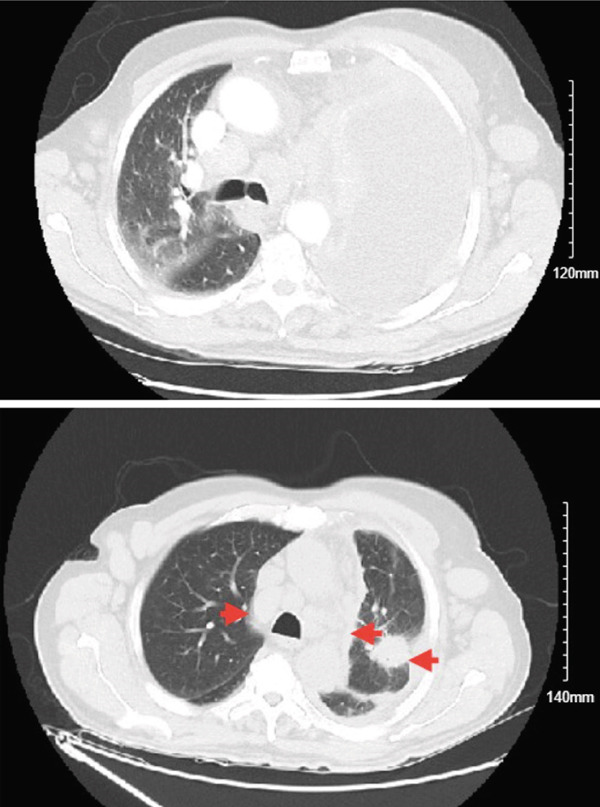
Chest CT showed a large left pleural effusion and revealed bulky bilateral axillary, right paratracheal, subcarinal, and right hilar lymphadenopathy. A left upper lobe mass of 3.3 × 4.3 cm was revealed after drainage of pleural effusion. Red arrows on the right are pointing to the borders of the left upper lobe mass, red arrow on the left is pointing to the bulky right mediastinal adenopathy.

Initial chest radiograph noted total opacification of the left hemithorax with a shift of the mediastinum to the right side (Figure 1). A chest CT confirmed a large left pleural effusion and revealed bulky bilateral axillary and right intrathoracic lymphadenopathy (Figure 2). A left‐side thoracentesis was done on two separate occasions, removing a total of 3.9 L of turbid bloody fluid. Pleural fluid analysis showed WBCs of 10,450/*μ*L with 99% lymphocytes, a few mesothelial cells, LDH 466 U/L, total protein 5.3 g/dL, albumin 2.6 g/dL, and adenosine deaminase 36 U/L (Ref 0–30 U/L). Fluid bacterial and fungal cultures were negative. Repeat CT chest 2 days after drainage exposed a 3.1 cm spiculated mass in the left upper lung lobe as well as low‐density metastatic lesions scattered within the liver and multiple lytic bone lesions suspicious for bone metastases. The cytology from the pleural fluid was consistent with adenocarcinoma of primary lung origin and CLL. Peripheral blood cytometry confirmed the diagnosis of CLL (90%) with CD5 and CD23 expressions (Table 1). The patient underwent a CT‐guided biopsy of the lung mass 5 days after the initial presentation, which confirmed the diagnosis of lung adenocarcinoma with PD‐L1 < 1% and EGFR alteration 16% (Figure 3).

**Table 1:** Biogenetic characteristics of lung cancer and CLL in Cases 1 and 2.

	**Case 1**	**Case 2**
Lung cancer	Positive mutations: EGFR 16% Negative mutations: AKT1, ALK, ATR, BRAF, CHEK1, DDR2, ERBB2, ERBB3, FGFR1, KRAS, MAP2K1, MET, NRAS, NTRK1, PIK3CA, POLD1, POLE, ROS1, STK11, TERT, TP53	Positive mutations: KRAS p.(Gly12Cys), KEAP1 p.(Glu446TER), KEAP1 p.(Gly477Val), NTRK1 p.(Asp509His) Negative mutations: ATK1, ALK, ATR, BRAF, CDKN2A, CHEK1, DDR2, EGFR, ERBB2, FGFR1, HRAS, MAP2K1, MET, NRAS, PIK3CA, POLD1, POLE, ROS1, SMARCA4, STK11, TERT, and TP53
CLL	B‐cell population 90% Positive CD5 and CD23 Positive ZAP70 Dim kappa Dim CD20, Negative CD38 *Karyotype*: 46,XY, no evidence of chromosomal abnormality IGVH hypermutation could not be interpreted Negative Monosomy 13 and Trisomy 12 FISH: Positive deletion of DLEU1, DLEU2 on Chromosome 13q14 Negative: t(11; 14)/CCND1‐IGH rearrangement, deletion of ATM on the long arm of Chromosome 11 at q22, and TP53 deletion on the short arm of Chromosome 17 at p13	B‐cell population 10% Positive CD5 and CD23 Negative kappa Dim lambda Negative CD20 and CD38 *Karyotype*: 46,XX with t(7; 13)(q32; q14) IGVH somatic hypermutation 9.6% Negative Monosomy 13 and Trisomy 12 FISH: Positive deletion of DLEU1 and DLEU2 on 13q14 Negative: t(11; 14)/CCND1‐IGH rearrangement, deletion of ATM on the long arm of Chromosome 11 at q22, and TP53 deletion on the short arm of Chromosome 17 at p13

Figure 3Biopsy from left lung lesions showing lung adenocarcinoma: (a) hematoxylin and eosin stain high‐power microscopic view, (b) hematoxylin and eosin stain low‐power microscopic view, (c) Napsin A staining, (d) TTF1 staining, (e) CD20 staining, and (f) CD23 staining.

**Figure:**
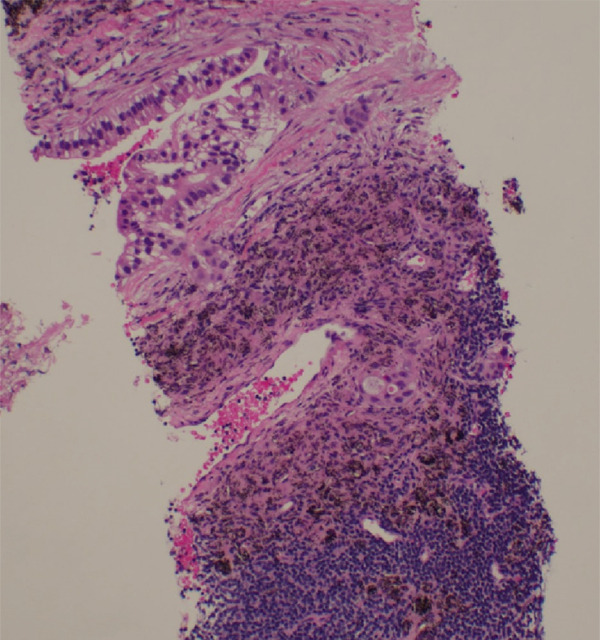
(a)

**Figure:**
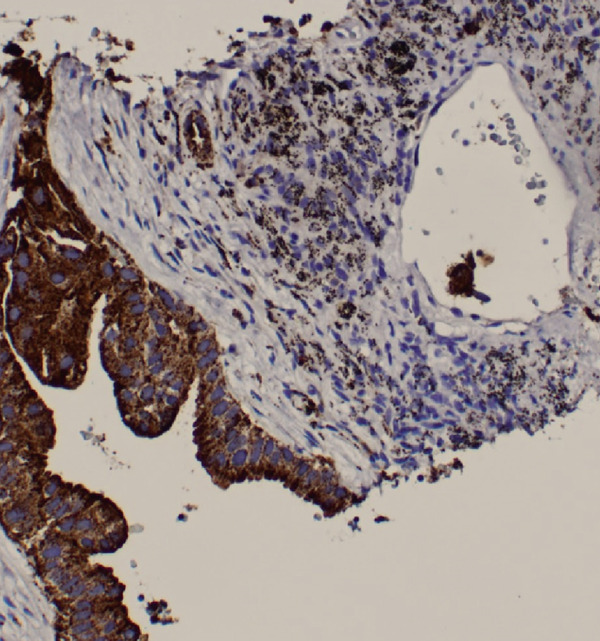
(b)

**Figure:**
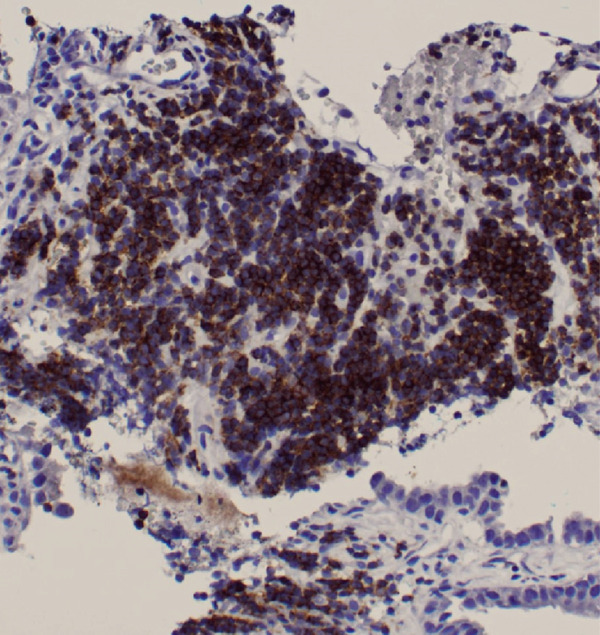
(c)

**Figure:**
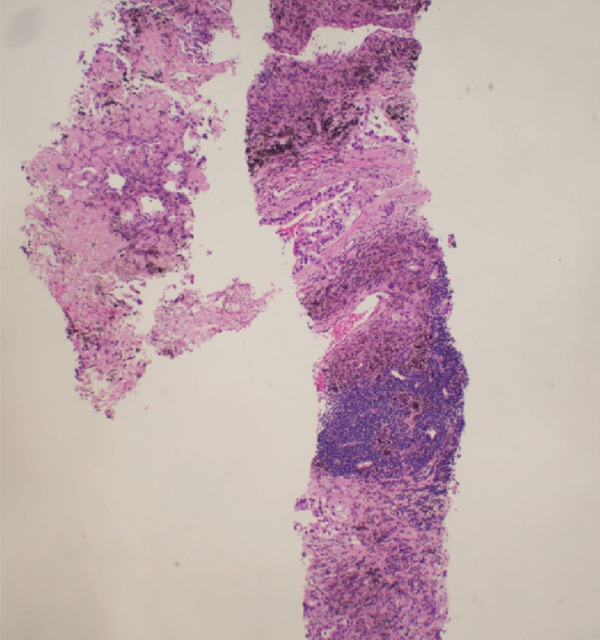
(d)

**Figure:**
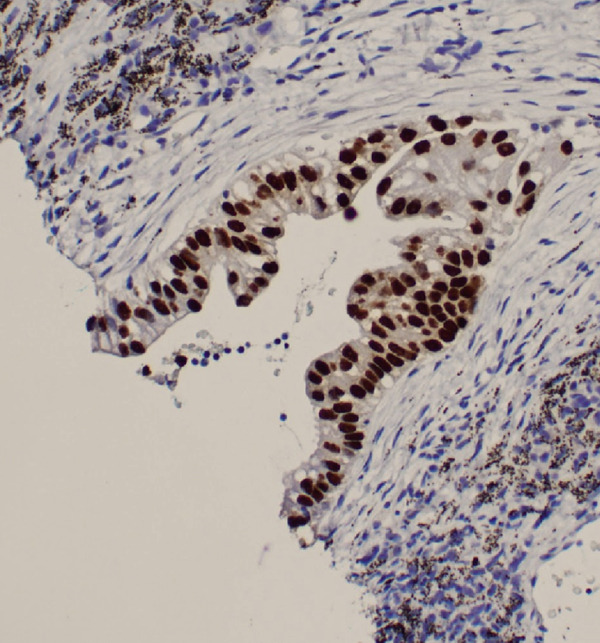
(e)

**Figure:**
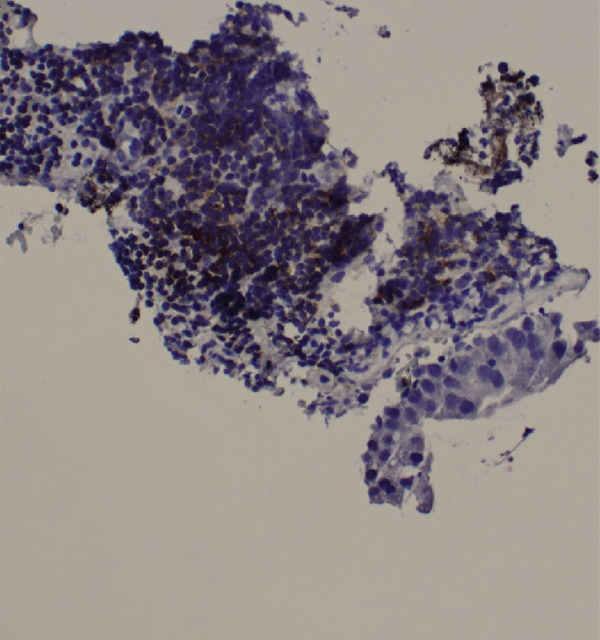
(f)

**Figure 4:**
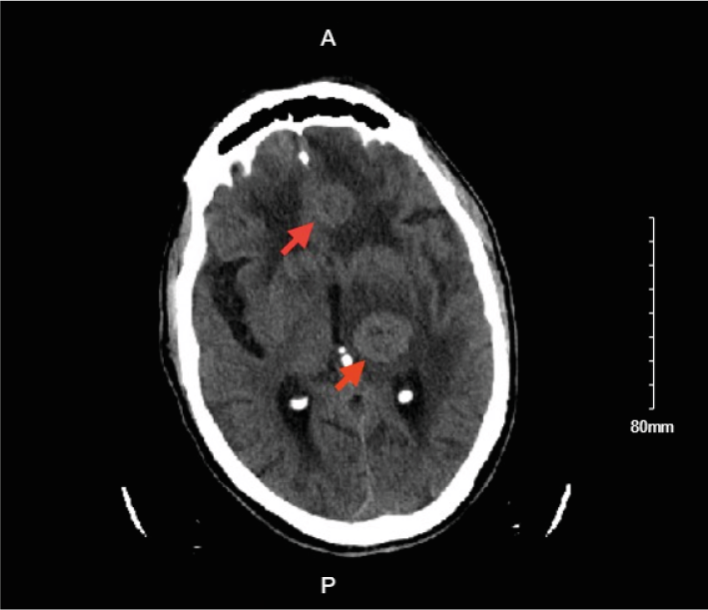
CT head showing acute hemorrhagic metastasis in the left frontal cortex, the high right frontal, and the left thalamus measuring 1.5 × 1.6, 0.5 × 0.6, and 1.8 × 2 cm, respectively, surrounded by vasogenic edema. Red arrows are pointing to the hemorrhagic brain metastasis.

The patient was discharged with a plan to start osimertinib given the EGFR mutation; however, a month later, he presented to the emergency department with altered mental status. CT head showed multiple hypodensities in the left frontal cortex, the high right frontal, and the left thalamus measuring 1.5 × 1.6, 0.5 × 0.6, and 1.8 × 2 cm, respectively, surrounded by vasogenic edema and described as acute hemorrhagic metastases (Figure 4). The patient was intubated and died the next day.

### 2.2. Case 2

A 52‐year‐old female with a history of 36 pack‐year smoking presented with progressive dyspnea on exertion for 1 month associated with right upper extremity numbness and weakness, blurring of vision, and unintentional weight loss of 13 lb. Her physical exam was remarkable for regular tachycardia with a heart rate of 110, cachexia, moderate respiratory distress, voice hoarseness, diminished breath sounds on the right upper lung with good air entry on other lung fields, and pain to light touch on the right upper back and shoulder. Initial labs showed WBCs of 43.8 × 10^3^/*μ*L, absolute neutrophil count of 36.5 × 10^3^/*μ*L, absolute lymphocyte count of 5.3 × 10^3^/*μ*L, hemoglobin of 10.7 g/dL, RBCs of 3.6 × 10^3^/*μ*L, MCV of 93.1 fL, platelets of 477 × 10^3^/*μ*L, and D‐dimer of 458 ng/mL.

A chest radiograph showed right upper lobe opacity. CT chest angiogram ruled out pulmonary embolism and showed an infiltrating mass entirely replacing the right upper lung lobe with additional pulmonary nodules bilaterally, large mediastinal and hilar lymphadenopathy, and a loculated right pleural effusion (Figure 5). Multiple scattered hepatic metastases were also noted. CT head was done for staging, showing a 2.2 cm enhancing mass in the left frontal lobe with moderate adjacent vasogenic edema, a 0.2 cm left‐to‐right midline shift, and a smaller lesion in the superior left precentral gyrus. A thoracentesis was done, draining 400 mL from the right pleural effusion, and fluid cytology was sent. A biopsy from the right supraclavicular lymph node was also obtained showing metastatic carcinoma with high tumor mutation burden 13.3 mutations/MB, microsatellite instability negative, KRAS p.(Gly12Cys), KEAP1 p.(Glu446TER), KEAP1 p.(Gly477Val), and NTRK1 p.(Asp509His) (Figure 6).

Figure 5(a) Large mass infiltrating the right upper lung lobe with large loculated right pleural effusion and associated hilar adenopathy, (b) large right infiltrating mass with complete consolidation of the right upper and patchy consolidation of the right middle and lower lung lobes, and (c) large area of parenchymal hemorrhage within the left cerebral frontal lobe with mixed low and increased attenuations, surrounding vasogenic edema and midline shift toward the right.

**Figure:**
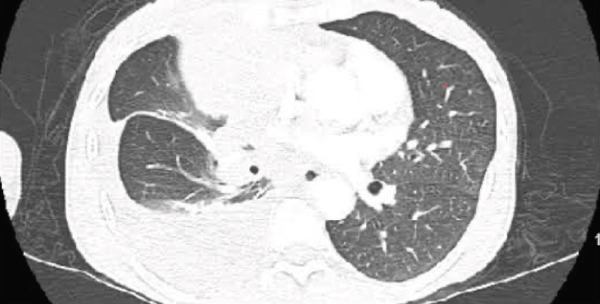
(a)

**Figure:**
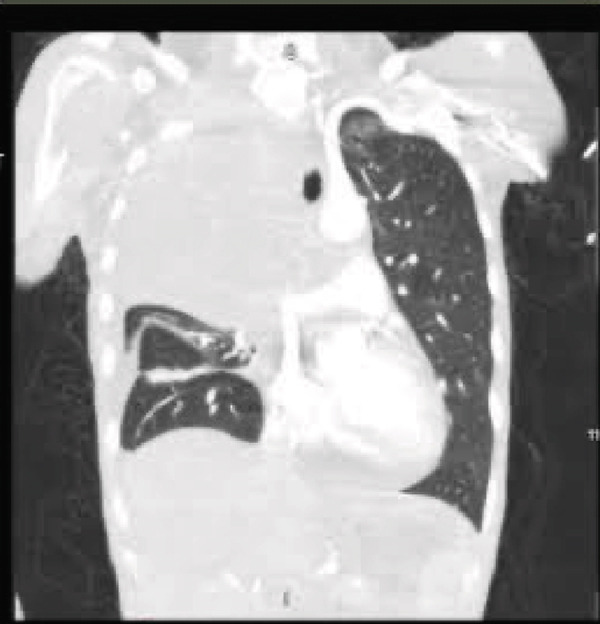
(b)

**Figure:**
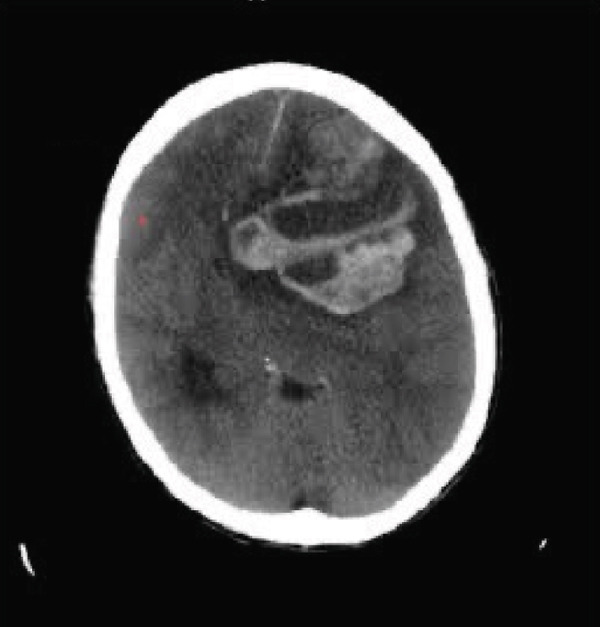
(c)

Figure 6Right supraclavicular mass biopsy showing metastatic carcinoma: (a) hematoxylin and eosin low‐power microscopic view, (b) hematoxylin and eosin high‐power microscopic view, (c) cytokeratin CAM 5.2 staining, and (d) cytokeratin AE1/E3 staining.

**Figure:**
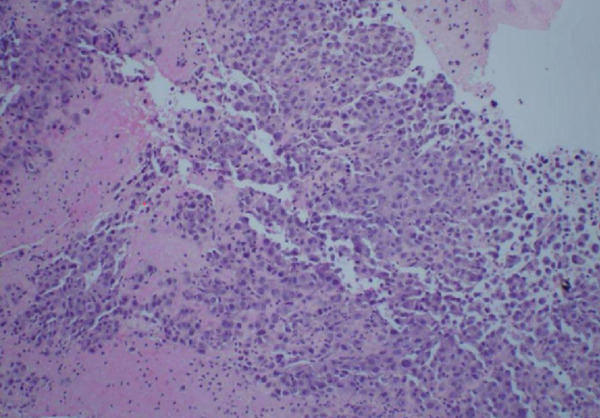
(a)

**Figure:**
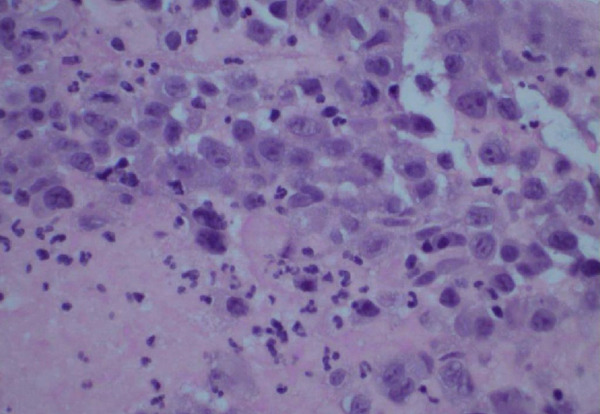
(b)

**Figure:**
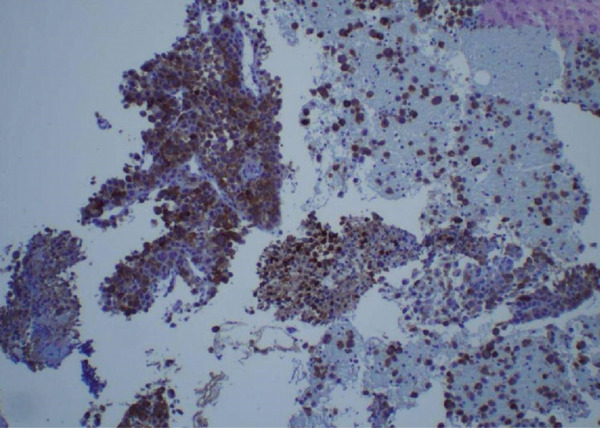
(c)

**Figure:**
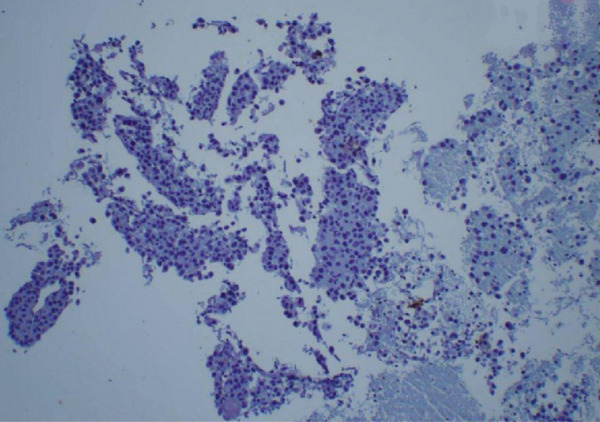
(d)

Although the patient′s leukocytosis was neutrophil‐predominant, flow cytometry was positive for CLL with small clonal lambda, positive CD5, and CD23. The B‐cell population is 10% comprising 8.6 K/*μ*L. Genetic analysis showed deletion of DLEU1 and DLEU2 genes on 13q14, IGVH somatic hypermutation 9.6%, and cell karyotype 46,XX with t(7; 13)(q32; q14), negative Monosomy 13 and Trisomy 12.

The patient was started on radiation therapy, with chemotherapy to follow; however, the hospital course was complicated with pneumonia and ended with a large left frontal lobe intracerebral hemorrhage within the brain metastasis resulting in massive vasogenic edema and death only 1 month after her initial diagnosis.

## 3. Discussion

CLL, the exact etiology of which continues to remain enigmatic, is an indolent malignancy that can be defined as a monoclonal lymphoproliferative disease characterized by the proliferation and accumulation of morphologically mature but immunologically dysfunctional B lymphocytes [4]. Accounting for 25%–30% of total leukemias in the United States, the disease manifests predominantly with immunologic, hematologic, and reticuloendothelial clinical signs [5].

The incidence of second primary malignancies (SPMs) is reported to be higher in patients with CLL compared to the general population, impacting morbidity and worsening survival [1]. Development of a third malignancy was also reported to be more common when lung cancer and CLL coexist [6].

We present two cases with both CLL and lung cancer diagnosed simultaneously with unique presentations and similar outcomes, to explore the mutual relation, risks for both malignancies′ coexistence, and characteristics of the patient population at increased risk of developing a second lung malignancy in CLL.

As proposed by Warren and Gates, multiple primary malignancies, in other words, synchronous or metachronous primary cancers, are defined by the following three conditions: (a) Each tumor must be histopathologically malignant, (b) the tumors must occur in different parts or organs, and (c) metastasis must be excluded [7]. In our cases, both CLL and the lung primary were proven to be histopathologically malignant through flow cytometry and CT‐guided lung biopsy, respectively, and interestingly, in the first case, the two tumors contributed to the pathology of the pleural effusion. As to the question of whether the two primaries were synchronous or metachronous, the timeline of both tumors before diagnosis is unclear, and it is uncertain if the second lung primary developed in the first 6 months in the course of CLL to be deemed as synchronous as opposed to metachronous.

Lung cancer is among the malignancies associated with CLL as documented in several reports in the literature since the 70s. Perhaps, two of the largest cohorts of CLL patients are those studied by Greene et al. and Travis et al., who reported increased incidences of lung cancer, brain cancer, soft tissue sarcomas, and malignant melanoma in CLL patients. The authors also referred to the fact that the cancer risk was constant across all time intervals after the diagnosis of CLL [8, 9]. A statistically significant increase in the relative risk of second lung cancer in CLL patients was identified in the retrospective registry‐based cohort study conducted in Denmark in 2005, which included a total of 9541 patients with CLL identified between 1943 and 1999 [3].

In terms of lung cancer pathology in CLL patients, both non–small cell and small cell lung cancer may develop; adenocarcinoma however is the most commonly documented as seen in the first case [10].

Many groups have undertaken the endeavor to explore the causal relationship between CLL and the development of a second primary. In a study carried out at Memorial Sloan Kettering Cancer Center between 1977 and 1998, out of the 1329 patients with CLL, approximately 2% of the patients developed lung cancer, and 85% of those patients were smokers. They also reported that lung carcinoma in patients with CLL was usually diagnosed after a decade and that the cause of death in these patients is usually lung carcinoma complications rather than CLL or other solid tumors [2]. A significant shared factor between the cases identified was tobacco smoking; however, the exact association has not been established. Another potential causal relationship that was proposed is the hereditary predisposition in cases demonstrating familial aggregation as genetic polymorphisms in enzymes metabolizing carcinogens which may dictate increased individual susceptibility to malignant diseases [3]. Both of our patients share the risk of smoking history as a mutual risk factor for both malignancies.

Additionally, the literature demonstrates significant immunological impairment in the pathology of CLL which involves all of the cellular components of the immune system, including quantitative and qualitative aspects of the normal B‐cell pool, T‐cell subsets, natural killer cells, and dendritic cells. These defects can impair the antigen‐presenting capacity of CLL B cells, thereby rendering the T cells anergic and predisposing for the development of second malignancies [11–13].

In a report of two cases of concurrent CLL/SLL and lung cancer, exploring the common genetic association, both CLL had Trisomy 12, and both lung tumors had high PD‐L1 expression and negative ALK mutation. Unlike cases reported by Nakao et al., only one of our cases had an EGFR mutation in lung cancer, and both of them lacked PD‐L1 expression as well as lack of Trisomy 12 in CLL [14].

The retrospective study by Chatzikonstantinou et al. pointed to the higher prevalence of lung cancer with CLL that has unmutated IGHV with an odds ratio of 2.09 (1.07–4.09) and a *p* value of 0.03 [15].

In a more recently published retrospective analysis conducted on 38 patients with coexisting CLL and lung cancer, nearly 40% of CLL had unmutated IGHV and del13q and, to a lesser extent, del11q and Trisomy 12 [6]. Nearly half of CLL in this analysis were in the early stage, while the other half required CLL‐directed treatment. The same analysis reported the common features of the associated lung cancer, including the adenocarcinoma pathology and at least one driver mutation in nearly half of the cases, including KRAS, EGFR, ALK, BRAF, and ATM [6].

Other proposed lung cancer biogenetic markers are HER‐2/neu overexpression which is identified as a significant contributor to the development and progression of lung cancer in patients with CLL [16]; this is in contrast to our cases, whose histological analysis of the lung mass was negative for HER‐2/neu overexpression.

It is worth pointing out that although it is rarely studied in literature, there appears to be no clear risk for CLL‐directed treatment on the lung cancer development and progression, which has been reported in the retrospective analysis by Chatzikonstantinou et al. [15]. On the contrary, immunotherapy directed to lung cancer might have a potential effect on CLL as a disease of the immune system. This is demonstrated in one of the cases reported by Nakao et al. where there was a suggested potential impact of lung cancer treatment with ipilimumab and nivolumab on the rapid progression of CLL [15].

Overall, both diseases are common, heterogeneous, and share a common ground and risk factors, which impact treatment and survival. For both of our patients, the outcome was similarly grave with a 1‐ and 2‐month survival from the time of diagnosis, reflecting the highly aggressive lung disease which usually has a higher impact on mortality than CLL.

## 4. Conclusions

The causal relationship between CLL and lung cancer has not yet been established. Given the increased yearly literature reports, the coexistence of both malignancies is worth exploring. Identifying and treating the SPMs may play a pivotal role in enhancing survival among patients with CLL, particularly given its generally indolent course. Numerous studies have highlighted the prevalence of SPMs such as lung cancer in CLL patients. Smoking remains the primary association of the highest incidence. An immunocompromised state in CLL patients is a notable risk factor. The biogenetic relationship and common characteristics between cases, however, are not fully identified. Further exploration is warranted to elucidate this relationship focusing on identifying potential therapeutic targets and assessing the potential increase in survival benefits for these patients.

**Nomenclature**
CLL: chronic lymphocytic leukemia
SPMs: second primary malignancies

## Consent

Written informed consent for the publication of this case report and any accompanying images was obtained from the patients′ family members.

## Conflicts of Interest

The authors declare no conflicts of interest.

## Funding

No funding was received for this manuscript.

## Data Availability

The data that support the findings of this study are available on request from the corresponding author. The data are not publicly available due to privacy or ethical restrictions.
